# Preventive Effect of Cow’s Milk Fermented with *Lactobacillus paracasei* CBA L74 on Common Infectious Diseases in Children: A Multicenter Randomized Controlled Trial

**DOI:** 10.3390/nu9070669

**Published:** 2017-06-27

**Authors:** Giovanni Corsello, Maurizio Carta, Roberto Marinello, Marina Picca, Giulio De Marco, Maria Micillo, Dante Ferrara, Patrizia Vigneri, Gaetano Cecere, Pasqualina Ferri, Paola Roggero, Giorgio Bedogni, Fabio Mosca, Lorella Paparo, Rita Nocerino, Roberto Berni Canani

**Affiliations:** 1 Operative Unit of Pediatrics and Neonatal Intensive Therapy, Mother and Child Department, University of Palermo, 90121 Palermo, Italy; giocors@alice.it (G.C.); cxbn@unin.it (M.C.); 2 Federazione Italiana Medici Pediatri Lombardia, 46100 Mantova, Italy; hetht@unin.it; 3 Pediatric Society of Primary Health Care (SICuPP), 20135 Milan, Italy; dafnfhm@unin.it; 4 Department of Translational Medical Science—Pediatric Section, University of Naples “Federico II” Via S. Pansini, 5, 80131 Naples, Italy; gntm@unin.it (G.D.M.); csvbu@unin.it (M.M.); rtntm@unin.it (G.C.); ryhm@unin.it (P.F.); paparolorella@gmail.com (L.P.); ritanocerino@alice.it (R.N.); 5 Department of Sciences for Health Promotion and Mother and Child Care, University of Palermo, 90121 Palermo, Italy; uhvbu@unin.it; 6 Family Pediatrician, 90133 Palermo, Italy; vignerip@gmail.com; 7 Department of Clinical Science and Community Health, Neonatal Intensive Care Unit, Fondazione I.R.C.C.S. Cà Granda Ospedale Maggiore Policlinico, University of Milan, 20143 Milan, Italy; sdvedd@unin.it (P.R.); fabio.mosca@mangiagalli.it (F.M.); 8 Clinical Epidemiology Unit, Liver Research Center, Basovizza, 34012 Trieste, Italy; giorgiobedogni@gmail.com; 9 European Laboratory for the Investigation of Food-Induced Diseases, University of Naples “Federico II”, 80131 Naples, Italy; 10 CEINGE Advanced Biotechnologies, University of Naples “Federico II”, 80131 Naples, Italy

**Keywords:** acute gastroenteritis, upper respiratory tract infections, probiotics, innate immunity, acquired immunity, gut microbiota, immunonutrition

## Abstract

**Background:** Fermented foods have been proposed to prevent common infectious diseases (CIDs) in children attending day care or preschool. Objectives: To investigate the efficacy of dietary supplementation with cow’s skim milk fermented with the probiotic *Lactobacillus paracasei* CBA L74 in reducing CIDs in children attending day care or preschool. **Methods:** Multicenter, randomized, double-blind, placebo-controlled trial on healthy children (aged 12–48 months) consuming daily 7 grams of cow’s skim milk fermented with *L. paracasei* CBA L74 (group A), or placebo (maltodextrins group B) attending day care or preschool during the winter season. The main outcome was the proportion of children who experienced ≥1 episode of CID during a 3-month follow-up. Fecal biomarkers of innate (α- and β-defensins, cathelicidin) and acquired immunity (secretory IgA) were also monitored. **Results:** A total of 126 children (71 males, 56%) with a mean (SD) age of 33 (9) months completed the study, 66 in group A and 60 in group B. At intention to treat analysis, the proportion of children presenting ≥1 CID was 60% in group A vs. 83% in group B, corresponding to an absolute risk difference (ARD) of −23% (95% CI: −37% to −9%, *p* < 0.01). At per-protocol-analysis (PPA), the proportion of children presenting ≥1 CID was 18% in group A vs. 40% in group B, corresponding to an absolute risk difference (ARD) of −22% (95% CI: −37% to −6%, *p* < 0.01). PPA showed that the proportion of children presenting ≥1 acute gastroenteritis (AGE) was significantly lower in group A (18% vs. 40%, *p* < 0.05). The ARD for the occurrence of ≥1 AGE was −22% (95% CI: −37% to −6%, *p* < 0.01) in group A. Similar findings were obtained at PPA regarding the proportion of children presenting ≥1 upper respiratory tract infection (URTI), which was significantly lower in group A (51% vs. 74%, *p* < 0.05), corresponding to an ARD of −23% (95% CI: −40% to −7%, *p* < 0.01). Significant changes in innate and acquired immunity biomarkers were observed only in subjects in group A. **Conclusions:** Dietary supplementation with cow’s skim milk fermented with *L. paracasei* CBA L74 is an efficient strategy in preventing CIDs in children.

## 1. Introduction

Respiratory and gastrointestinal tract infections are a relevant problem for young children attending day care or preschool, especially in the winter season. These conditions, termed common infectious diseases (CIDs), are facilitated by a general immaturity of the immune system and of respiratory and gastrointestinal tract functions [1]. The frequency of these infections cause significant discomfort for children and their parents, as well as costs in terms of medical examinations, use of drugs, need for hospitalization, and school and working days lost by the parents [1]. The prevention of CIDs in young children is therefore of major importance. One option is to use fermented foods [2]. Actually, 90% of naturally fermented foods in different countries are still produced at home using traditional procedures [2]. Fermented foods could contain both functional and non-functional microorganisms [3]. Among the functional features of microorganisms in fermented foods are probiotic properties [4], antimicrobial properties [5], antioxidants [6], and bioactive peptides production [7], which are relevant criteria for the selection of starter cultures to be used in the preparation of functional foods [8]. Evidence suggests that functional foods derived from the fermentation of cow’s milk with probiotic strains could prevent infectious diseases in children, but data are still conflicting [9,10,11,12,13,14,15]. Differences in the experimental design, study populations, and in bacterial strains used in the preparation of the fermented products could be responsible for these discrepancies [9,10,11,12,13,14,15]. A recent double-blind randomized controlled trial (RCT) has shown that cow’s skim milk fermented with *Lactobacillus paracasei* CBA L74 is effective at preventing CIDs in children aged 12–48 months attending day care or preschool. In detail, the children consuming the fermented milk had a lower incidence of the respiratory and gastrointestinal tract infections compared to the control group. This effect was associated with a significant stimulation of innate and acquired immunity [16]. In order to confirm these results, we performed a multicenter RCT to evaluate the efficacy of cow’s skim milk fermented with *Lactobacillus paracasei* CBA L74 at reducing CIDs in healthy children attending day care or preschool during the winter season.

## 2. Material and Methods

### 2.1. Study Design

A multicenter randomized, double-blind, placebo-controlled trial was performed between December 2014 and March 2015 in collaboration with family pediatricians (FPs) operating in Palermo, Milan, and Naples. Participants follow-up was performed from 20 December 2014 to 20 March 2015.

The FPs care for children up to 14 years of age in the Italian public health system. The study was approved by the Ethics Committee of the Universities of Palermo, Milan and Naples, and it was registered in the Clinical Trials Protocol Registration System (ClinicalTrials.gov) with the identifier NCT02367612. Before the start of the study, all FPs attended two investigator meetings during which the study protocol was illustrated and discussed, and all procedures and definitions were shared.

### 2.2. Study Subjects

Healthy children aged 12–48 months, attending day care or preschool for at least 5 days a week, regularly checked by the FPs involved in the trial, were considered for the study and consecutively contacted during scheduled medical examinations at the FPs office. The FPs consulted the clinical records of each child for previous diseases and pharmacological treatments. At the baseline, after obtaining informed consent from the parents/tutors of each child, the health status of all the study subjects was carefully assessed, and the presence of infectious diseases or other disease was ruled out by means of a complete physical examination, including vital signs (body temperature, pulse rate, respiration rate, blood pressure); neurological status; body growth status; nutritional status; hydration; skin evaluation; otoscopy; evaluation of oral cavity; respiratory/abdomen/lymphonode examination; and genital examination. The exclusion criteria were age <12 months or >48 months, concomitant chronic infections, chronic systemic diseases, chronic inflammatory bowel diseases, autoimmune diseases, immunodeficiency, malignancy, metabolic diseases, chronic respiratory tract diseases including respiratory allergies and cystic fibrosis, malformations of gastrointestinal or urinary or respiratory tract, history of respiratory or gastrointestinal or urinary tract surgery, congenital cardiac defects, functional bowel disorders, suspected or challenge-proved food allergy, food intolerances, severe malnutrition (z-score for weight-for-height <3 standard deviation scores), and use of antibiotics or pre/pro/synbiotics or immune stimulating products in the 2 weeks before the enrolment. Siblings of subjects enrolled in the study were not allowed to participate to the trial. Anamnestic, demographic, and clinical data, including vaccination status, were collected by the FPs and reported into a specific form.

### 2.3. Intervention

The investigators were blinded to the treatment at all times (i.e., allocation, intervention, laboratory analysis and statistical analysis). The study subjects were allocated to two groups (group A or group B) according to a computer-generated randomization list. The FPs, parents, and the children were blinded to the allocated treatment. Subjects were supplemented daily for 3 months with cow’s skim milk fermented with *L. paracasei* CBA L74 (group A) or placebo (group B). Table 1 reports the composition of the study dietary products.

Participants were supplied with the milk product in powder form, by Heinz Italia SpA, Latina, Italy, an affiliate of the Kraft Heinz Company, co-headquartered in Pittsburgh, PA, and Chicago, IL, USA. The fermented product was prepared with a standard fermentation process. Briefly, cow’s skim milk powder was mixed with sterile water and then exposed to Ultra High Temperature (UHT) treatment. Fermentation was started in the presence of 10^6^ bacteria (*L. paracasei* CBA L74), reaching 5.9 × 10^9^ colony-forming units/g after a 15 h incubation at 37 °C. *L. paracasei* CBA L74 was added as fresh cultures. The initial pH was 6.6. Lactobacilli selective agar (LBS) was used for detection of *L. paracasei* CBA L74. *L. paracasei* CBA L74 was incubated at 37 °C anaerobically. Plate count agar (PCA) was used for detection of contaminants.

After heating at 85 °C for 20 s in order to inactivate the live bacteria, the product was spray-dried. Thus, the final fermented milk powder contained only bacterial bodies and fermentation products and no living microorganisms. The placebo consisted of maltodextrins with an energy content similar to that of the fermented milk. With the goal of avoiding any possible influence on the stool pattern of children participating into the study, the placebo composition was designed in order to provide no more than 2.9 g/day of maltodextrins. The study products were provided in tins containing 400 g of powder, and the packaging was similar and the tins were stored at room temperature in a dry environment. The FPs instructed parents about the daily amount of the assigned study product and the method of preparation. All subjects received 7 g/day of study products diluted in a maximum of 150 mL of cow’s milk or water. After dilution, the look and the taste were the same for all of the study products. The parents were instructed to contact the FPs if necessary and to maintain the habitual diet of the child, and to avoid prebiotics, probiotics, synbiotics, and immune stimulating products during the 3-month study period. During episodes of acute gastroenteritis (AGE), or other morbidities, children were instructed to continue the assigned study product.

### 2.4. Study Monitoring

Study monitoring was performed by an independent RCT monitor, blinded to the treatment assignment. Study monitoring included on-site medical examinations and telephone communications with FPs, to ensure that the investigation was conducted according to the protocol. The clinical trial monitor collected clinical forms; ensured compliance with the trial protocol; reviewed the clinical forms for completeness, clarity, and consistency.

### 2.5. Study Outcomes

The primary outcome of the trial was the rate of children experiencing at least one episode of CID. The secondary outcomes were: total number of CIDs, use of medications (antibiotics, antipyretics, steroids), emergency department medical examinations, hospitalizations, days of work lost by the parents, and days of school lost by the children.

Study groups were also compared for fecal levels of α- and β-defensins, cathelicidin (LL-37), and secretory immunoglobulin A (sIgA) at enrolment and after 3 months of intervention.

The possible occurrence of adverse events was also assessed.

### 2.6. Estimate of Sample Size and Randomization

Sample size was calculated taking into account the effect size estimated from a previous RCT [16]. We calculated that 55 children per group were needed to detect a change in the occurrence of at least one episode of CID from 79% in group A to 50% in group B with a power of 0.90 at an alpha level of 0.05 (Pearson’s Chi-square, two-tailed test). Assuming a dropout rate up to 25%, we calculated that 73 children per group had to be enrolled into the study. Randomization was based on a list with consecutive numbers with an allocation ratio of 1:1 between product A and B. Each treatment was numbered according to the randomization scheme without any reference to the group assignment, which was known only to the statistician who generated the list and to the technician who prepared the packages. The packages and content of treatments were indistinguishable. The software used to generate the randomization list was random.org (Randomness and Integrity Services Ltd., Dublin Ireland).

Five FPs per Center (Palermo, Milan and Naples) participated to the study. The study was considered terminated when the total number of calculated children was reached independently from the number of children enrolled by each center.

### 2.7. Data Collection

A diary was given to the parents by FPs, with instructions to report the following daily: systemic symptoms including fever, headache, restless, myalgia, irritability; gastrointestinal or respiratory symptoms; use of drugs (antibiotics, antipyretics, steroids); emergency department medical examinations; hospitalizations; possible adverse events; consumption of the study products; school days lost by the children; working days lost by the parents. The diary served as an indicator for the need of a medical examination and as a consistent way of recording and recalling symptoms.

For all the children, a medical examination was planned by the FPs at 30, 60 and 90 days, and whenever it was necessary because of infectious diseases or other morbidities. During these medical examinations, personal medical history and general clinical conditions of the children were evaluated, diaries were checked, the study products were provided to the parents for the next 4 weeks and the product tins were collected. Whenever symptoms of infectious diseases or other morbidities occurred, parents were instructed to contact the FPs to have a medical examination of their child. At these medical examinations, the FPs consulted the diary compiled by the parents, performed a full physical examination, and then, using standardized criteria, decided on the diagnosis (infectious origin: yes or no; if yes: gastrointestinal; or upper respiratory tract infection (URTI): rhinitis, tracheitis, laryngitis, pharyngitis, or acute otitis media; or other site).

### 2.8. Outcome Definitions

A diagnosis of acute gastroenteritis (AGE) was made in the presence of ≥3 bowel movements of soft/liquid stools in 24 h, with or without fever or vomiting, as indicated by the European Society for Pediatric Gastroenterology Hepatology and Nutrition (ESPGHAN) and the European Society for Pediatric Infectious Diseases (ESPID) [17]. The FPs were instructed to not diagnose antibiotic-associated diarrhea as AGE. Antibiotic-associated diarrhea was defined as diarrhea that occurs in relation to antibiotic treatment with the exclusion of other etiologies [18].

The diagnosis of URTI was made by FPs based on physical examination according with well defined standard criteria. As previously described, they could not be made without the occurrence of ≥1 acute respiratory symptom(s) (runny nose, cough, sore throat, aphony, shortness of breath, otalgia, otorrhea, extroversion of tympanic membrane with or without hyperemia) in the absence or presence of ≥1 systemic symptom(s) (fever, headache, restless, myalgia, irritability) [1,16,19,20]. Unspecific symptoms of infectious diseases such as fever, malaise, poor appetite, tiredness, restlessness, headache were not sufficient to make a diagnosis of URTI. In accordance with validated guidelines and common clinical practice, for the diagnosis, in all cases a cluster of signs and symptoms was evaluated by the FPs. More precisely, the criteria that the FPs used for the diagnosis of URTI, were the acute onset of the following signs and symptoms [21,22,23,24,25]:

- Rhinitis was defined by the presence of mucus secretion from the nose, nasal obstruction with or without sneezing, conjunctivitis, cough, and fever.
- Tracheitis was defined by symptoms of airway obstruction or impending respiratory failure or both (including cough, mucus production, shortness of breath, or fever).
- Laryngitis was defined by inspiratory wheezing with cough and hoarse voice with or without chest indrawing, stridor, aphony and fever.
- Pharyngitis was defined by inflammation of the pharyngeal tonsils that may be accompanied by other nonspecific symptoms (including cough, sore throat, and fever).
- Acute otitis media was defined by the presence of tympanic membrane inflammation and by the presence of ≥1 of the acute symptoms (otalgia, otorrhoea, irritability, and fever). The demonstration of tympanic membrane inflammation was based on the following otoscopic findings: (a) marked erythema with bulging and absence mobility due to the presence of middle ear effusion; (b) yellowish membrane by observing in transparency the presence of purulent material in the middle ear; or (c) presence of spontaneous perforation with otorrhoea. Redness alone of the tympanic membrane was considered insufficient for the diagnosis. In order to help the FPs in distinguishing acute otitis media from otitis media with effusion, a video provided by the American Academy of Pediatrics was also projected during the investigator meetings [26].

No infections were recorded in the absence of a physical examination performed by FPs involved in the study. All infectious diseases were recorded by FPs in a specific study form. Microbiological tests were performed only when required by specific clinical reasons. Compliance was defined as the consumption of at least 80% of the assigned treatment during the study and was evaluated by counting and weighing the returned tins and by reviewing the notes on the diary recorded by parents.

### 2.9. Assessment of Immunological Parameters

Stool samples (3 gr) were obtained at the start and end of the trial. Fecal samples were immediately frozen (−20 °C), brought frozen to the laboratory and stored at −80 °C. Fecal α-defensin, β-defensin, cathelicidin (LL-37), and sIgA were measured from the supernatants of fecal homogenates. Briefly, the homogenates were centrifuged (13,000× *g* per 10 min), and the supernatants were collected. For LL-37 measurement, the samples were extracted with 60% acetonitrile in 1% aqueous trifluoroacetic acid (TFA) and then extracted overnight at 4 °C, as described previously [27]. The extracts were centrifuged and their supernatants stored at −20 °C. HNP 1-3 was measured by ELISA using a human kit (Hycult biotechnology, Uden, The Netherlands); HBD-2by ELISA using a human kit (Phoenix Pharmaceuticals, Inc., Burlingame, CA, USA); LL-37 by ELISA using an human kit (Hycult biotechnology, Uden, The Netherlands), and sIgA by indirect enzyme immunoassay (Salimetrics LLC, Carlsbad, CA, USA). The results were expressed as ng/g for α-defensin (HNP 1-3), β-defensin (HBD-2), and cathelicidin (LL-37) and as μg/g of supernatant for sIgA.

### 2.10. Statistical Analysis

A biostatistician blinded to the treatment allocation performed the statistical analysis. Descriptive statistics are reported as means and standard deviations for continuous variables and as numbers and proportions for dichotomous variables. The main outcome was the occurrence of at least one episode of CID during the 3-month study period. Such an outcome was evaluated using intention to treat (ITT) analysis assigning the worst event, i.e., the occurrence of one CID, to the children with missing data. The absolute risk difference (ARD) for the occurrence of at least one CID in group A vs. group B was calculated using binomial regression. A secondary analysis added center as covariable to the binomial regression model (discrete: 2 = Palermo; 1 = Milan; 0 = Naples) to test whether it could impact the estimate of the effect size. As CID is a composite outcome, we also performed a per-protocol-analysis (PPA) on its components, i.e., URTI and AGE. A pre-specified secondary outcome was the incidence rate of CID in group A vs. group B. The incidence rate ratio (IRR) was evaluated under PPA using negative binomial regression instead of the pre-planned Poisson regression because of overdispersion. Other pre-specified outcomes were the changes in α-defensin, β-defensin, LL-37, and sIgA in group A vs. group B. Such changes were analyzed using random-effect linear regression. The response variable was the immunological marker (continuous) and the predictors were the baseline value of the response variable (continuous), time (discrete, 0 = baseline; 1 = 3 months), treatment (discrete, 0 = group B; 1 = group A), and a treatmentXtime (discreteXdiscrete) interaction. The treatmentXtime interaction gives a measure of the change in the immunological marker for group A vs. group B at 3 months vs. baseline. Immunological markers were log_e_-transformed to reduce skewness and ensure homoscedasticity of residuals. Statistical analysis was performed using SPSS 16.0 (IBM Corporation, Armonk, NY, USA) and Stata 14 (Stata Corporation, College Station, TX, USA).

## 3. Results

**Figure 1:**
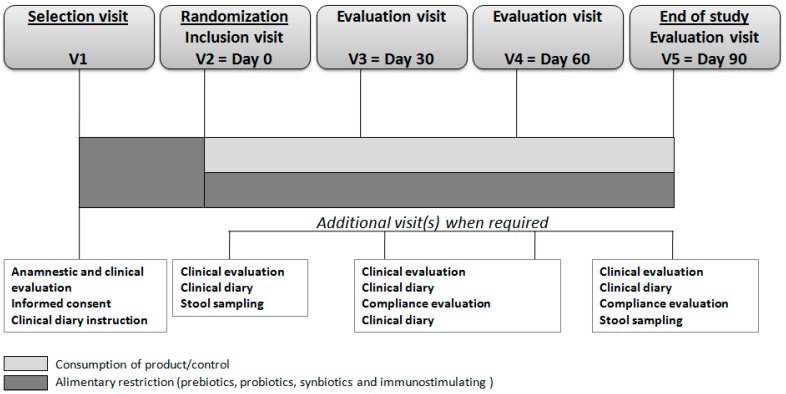
The design of the study.

**Figure 2:**
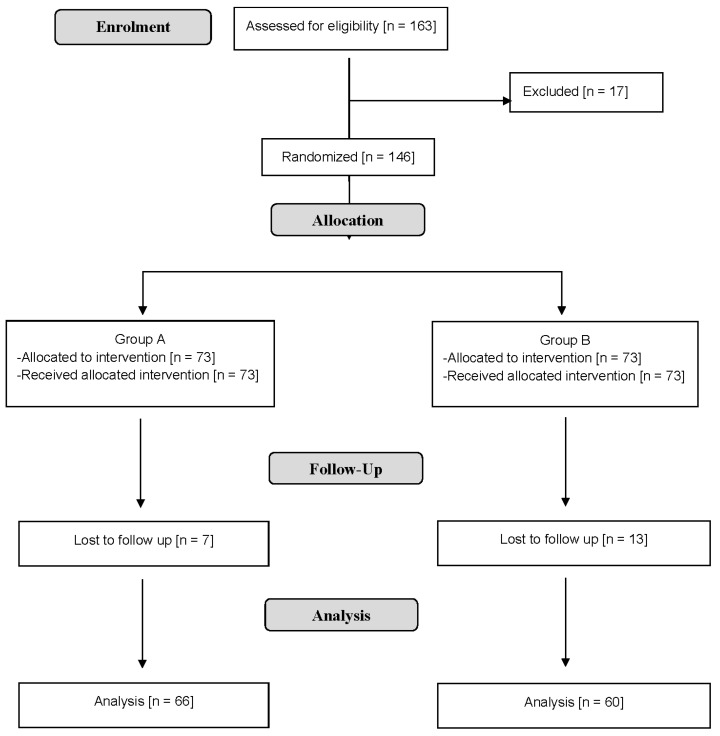
The flow of children through the study, Group A: Cow’s milk fermented milk with *L. paracasei* CBA L74; Group B: Placebo.

The study design is depicted in Figure 1. In Figure 2 is reported the flow of children during the study. A total of 146 children were enrolled into the trial: 73 in group A and 73 in group B.

No child refused to participate after randomization, and all children received the allocated intervention. Table 2 gives the main features of the children.

Main features of children enrolled in A and B groups were similar. All children were from families of middle socioeconomic status and were living in an urban area. All children were not febrile and free of infectious diseases at enrolment. The vaccination status, the breastfeeding rate and duration were similar among the two groups. No child had received anti-Rotavirus or anti-influenza vaccine. These vaccines were not usually included in the vaccination schedule of Italian healthy children.

The interventions were well accepted by the children and the overall compliance to them was good. A total of 20 subjects were lost to follow-up (7 in group A and 13 in group B). Thus, 126 children completed the study, 66 in group A and 60 in group B. No differences were detected in the daily intake of the active and placebo products. Also, no changes were observed for body weight and height in the two study groups, indicating that the consumption of the study products was safe, at least in the short term. No adverse events related to the consumption of the active or placebo products were recorded.

**Figure 3:**
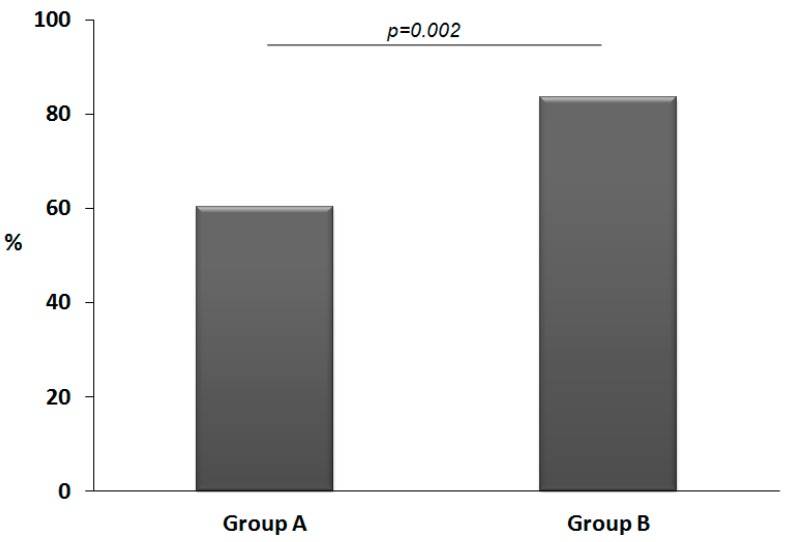
The proportion of children presenting at least one common infectious disease during the study period (intention-to-treat analysis). Group A: Cow’s milk fermented with *L. paracasei* CBA L74; Group B: Placebo. *p* = 0.002, chi-square test *p* < 0.01, absolute risk difference: −23% (95% CI: −37% to −9%, binomial regression).

The ITT analysis showed that during the trial, 105 out of the 146 (72%) children experienced at least one episode of CID. As showed in Figure 3, the proportion of children presenting at least one episode of CID was significantly lower in group A (60%) than in group B (83%). The corresponding ARD was −23% (95% CI: −37% to −9%, *p* < 0.01, binomial regression) for group A vs. group B. This absolute risk reduction (ARR) corresponds to a number of children needed to treat (NNT) of 4 (95% CI 3 to 11) for group A vs. group B.

Adding center as a covariable to the binomial regression model was not associated to any relevant change of the size of the effect of active treatment vs. placebo on CID (absolute risk change −0.24, 95% CI −0.40 to −0.08 without center vs. −0.23, 95% CI −0.38 to −0.07 with center), URTI (absolute risk change −0.23, 95% CI −0.40 to −0.07 without center vs. −0.23, 95% CI −0.39 to −0.07 with center) and AGE (absolute risk change −0.22, 95% CI −0.37 to −0.06 without center vs. −0.24, 95% CI −0.39 to −0.09 with center). At PPA, the rate of subjects presenting at least one episode of AGE was lower in group A (18%) than in group B (40%). The ARD for the occurrence of at least one episode of AGE was −22% (95% CI: −37% to −6%, *p* < 0.01, binomial regression) in group A compared to group B. At PPA, the proportion of children presenting at least one episode of URTI was 51% in group A and 74% in group B. The ARD for the occurrence of at least one episode of URTI in group A vs. group B was −23% (95% CI: −40% to −7%, *p* < 0.01, binomial regression). The incidence rate ratio (IRR) for CID, calculated under PPA, was 0.64 (95% CI: 0.42 to 0.98, *p* < 0.05, negative binomial regression) for group A vs. group B, corresponding to a mean (95% CI) number of CID of 2.8 (2.0 to 3.7) in group B vs. 1.8 (1.3 to 2.3) in group A. Table 3 gives the proportion of subjects who experienced at least one episode of rhinitis, otitis, pharyngitis, laryngitis, tracheitis or AGE.

Microbiological analyses for etiological investigations were not requested in any subjects.

**Figure 4:**
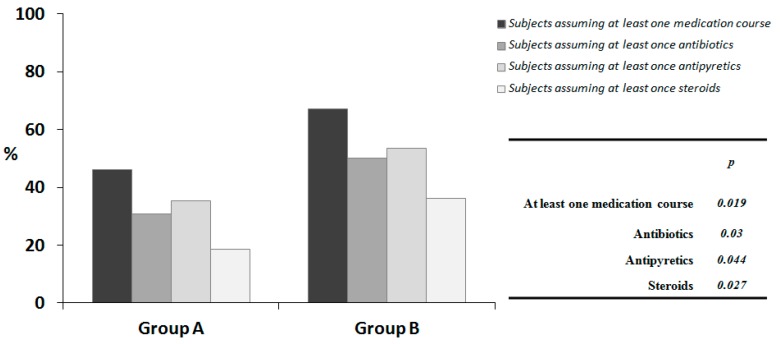
The proportion of children requiring medication use (i.e., antibiotics, antipyretics, steroids) during the study period. Group A: Cow’s milk fermented with *L. paracasei* CBA L74; Group B: Placebo.

At PPA, 45% of the children in group A received at least 1 medication course (antipyretics, antibiotics, or steroids) as compared with 65% of those in group B, corresponding to an absolute risk reduction of −20% (95% CI: −4% to −38%, *p* < 0.05, binomial regression, 3 subjects excluded from the analysis because of incomplete or missing data) (Figure 4).

During the study period, one child from group B required hospitalization for two days. At PPA, the IRR was 0.26 (95% CI 0.13 to 0.53, *p* < 0.001, negative binomial regression) for the count of lost days of school for group A vs. group B, corresponding to a mean (95% CI) of 2 (1 to 3) days for group A and 8 (4 to 12) days for group B. At PPA, the IRR was 0.18 (95% CI 0.07 to 0.47, *p* < 0.001, negative binomial regression) for the count of lost days of work of parents for group A vs. group B corresponding to a mean (95% CI) of 0.6 (0.2 to 1.0) days for group A and 3.3 (1.1 to 5.5) days for group B.

**Figure 5:**
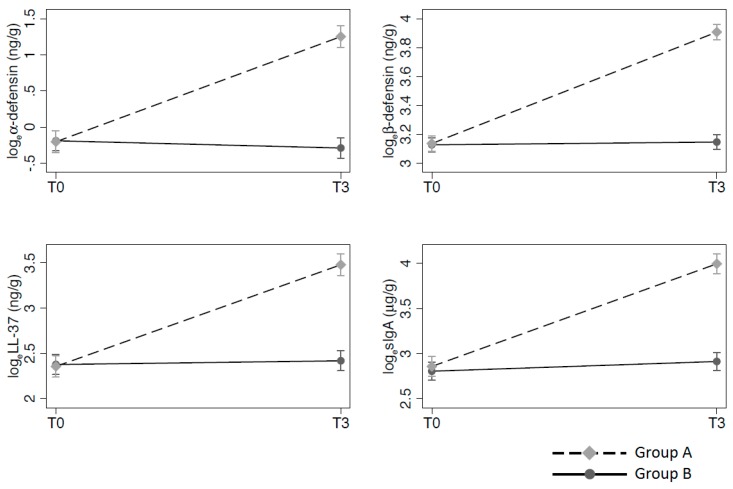
Determination of innate and acquired immunity biomarkers at enrolment and after 3-month treatment in children evaluated in the two study groups. Panel a: α-defensin; panel b: β-defensin; panel c: LL-37; panel d: sIgA. Values are means and 95% confidence intervals from random effect linear regression with correction for baseline. Group A: Cow’s milk fermented with *L*. *paracasei* CBA L74; Group B: Placebo.

Figure 5 plots the changes in fecal log_e_α-defensin, log_e_β-defensin, log_e_LL-37, and log_e_sIgA during the study. Significant changes in log_e_α-defensin, log_e_β-defensin, log_e_LL-37, and log_e_sIgA were seen at 3 months vs. the baseline for group A vs. group B.

## 4. Discussion

The results of this RCT confirm that dietary supplementation with cow’s skim milk powder fermented with *L. paracasei* CBA L74 is associated with a reduction of CIDs in young children attending daycare or preschool. This protective effect is accompanied by a reduction of medication use, working days, or days of school lost, and by a stimulation of innate and acquired immunity. The dietary supplementation was well accepted by the children and safe, as demonstrated by the low dropout rate together with the high level of adherence, and the absence of adverse events observed during the study period. Our inability to evaluate infectious episode etiologies in enrolled children represents a major limitation of the study. However, the strengths of our investigation include a multicentre enrolment, an adequate randomization and power to test the hypothesis, the use of a double-blind design, and a comprehensive follow-up strategy, all of which minimize the risk of bias.

The critical role of nutrition on the immune system is widely recognized, and we provided data on possible mechanisms of action elicited by this dietary product in protecting the children from CIDs.

The probiotic *L. paracasei* CBA L74 has been genetically characterized by the identification of its molecular profile using the Rep-PCR (Repetitive Extra-Genic Palindromic-PCR) technique, and it has been selected for its capacity to grow on milk. Data obtained in vitro on dendritic cells, ex vivo on intestinal biopsies, as well as in vivo and on a murine model of experimental colitis and of *S. typhimurium* infection showed that milk fermented with *L. paracasei* CBA L74 exerts anti-inflammatory properties in terms of stimulation of interleukin (IL)-10 production and reduction of IL-12 synthesis [28]. As an inactivated probiotic was used in this study, we confirm that the viability of the bacteria is not essential to exert an effect on the immune system [11,29].

On the contrary, differences in the bacterial strains used for the preparation of the fermented products could affect the immune function, because the immune response to probiotics is usually strain-dependent, even within the same species [4,9,10,11,12,13,14,15].

In the present study, we observed an immunostimulatory effect consisting of a significant increase of the production of innate and acquired immunity peptides. Innate immunity peptides, produced by epithelial cells, Paneth cells, neutrophils and macrophages, act as endogenous antimicrobial substances and defend the body against a broad range of pathogens (bacteria, fungi, protozoa, and viruses) [30]. In addition to their antimicrobial role, these peptides have been shown to regulate the activity of T cells, dendritic cells, macrophages, monocytes, and neutrophils [31]. In parallel, the production of sIgA, an important component of acquired immunity, was positively affected by the fermented dietary product. sIgAs not only play a pivotal role in local immunity being the first line of defense against pathogens in the mucosae, but also they regulate gut microbiota composition, driving the communication between gut commensal bacteria and the host immune system [32]. A positive effect on gut microbiota composition and function has been recently demonstrated in a human study, where a fermented milk product obtained from a mix of probiotic bacteria was able to increase the production of short chain fatty acids and decrease the number of pathobionts [33]. Intestinal IgA-producing B cells may migrate to other mucosal sites contributing to protection against pathogens. Indeed, it has been recently demonstrated that polyreactive IgA induced in the gut can facilitate the production of antigen-specific IgA both in feces and blood, thus inducing a generalized protection [34]. A further limitation of this study is derived by the lack of investigation on the expression of innate and acquired immunity biomarkers at respiratory tract level. Further studies are necessary to investigate the effect of this fermented product on immune and non-immune defense mechanisms against infection at respiratory and systemic level.

A Cochrane review concluded that probiotics are able to reduce the incidence of acute URTI and the frequency of antibiotic use and school absence [35]. Similar results have been reported using fermented dairy products containing Lactobacilli probiotics in pediatric as well in elderly populations [9,36,37]. It has been shown that the effects of fermented dairy products arise not only from cell wall components of the microorganism or from the cytoplasmic content (such as nucleotides), but also from peptides produced during the fermentation process [38]. These peptides could act as modulators of the immune system [39,40,41,42]. If confirmed in future studies, these findings will pave the way to new approaches in the use of probiotics added to different foods.

The favorable low number of children to treat and the net reduction in total number of infections observed in this study suggest that the use of milk fermented with *L. paracasei* CBA L74 could have relevant clinical, public health, and economic consequences. In addition, this fermented product does not contain living microorganisms. This feature limits the risk of bacterial translocation, and facilitates an easy storing of the product. Lastly, the activity of the fermented product does not depend on the in vivo fermentation process, which can vary widely according to subjects and conditions, as may be the case for prebiotic-containing formulas [43].

## 5. Conclusions

The results of this trial and those already proven [16], suggest that the use of milk fermented with *L. paracasei* CBA L74 could be a valid strategy in preventing CIDs in children attending educational programs. Such benefits are even more interesting at a time when the number of children attending daycare centers has increased all over the world [44] and traditional medicine seems not to be completely able to provide adequate response to the need for preventive strategy of disease using side-effect-free treatments. However, it is important to recognize that this RCT studied a specific fermented product with a specific probiotic strain, a well-defined dose, and age group, and that these findings cannot be extrapolated for other fermented products based on different probiotics strains.

## Figures and Tables

**Table 1:** The composition of the study dietary products.

Value for 100 g of Product	Group A Cow’s Milk Fermented with *Lactobacillus paracasei* CBA L74	Group B Placebo
Energy, kcal	367	388
Proteins, g	24.0	0
Carbohydrates, g	66.4	97
Fats, g	0.6	0
*Lactobacillus paracasei* CBA L74, CFU *	5.9 × 10^11^	-

* Killed bacteria.

**Table 2:** The main features of the study population at enrolment.

	Group A *n* = 73	Group B *n* = 73
Male, *n* (%)	39 (53.4)	45 (61.6)
Age, months (±SD)	32.5 (9.7)	33.7 (8.6)
Weight, kg (±SD)	14.8 (3.2)	15 (3)
Height, cm (±SD)	92 (8.1)	94 (7.6)
Breastfeeding, *n* (%)	50 (68.5)	41 (56.2)
Duration of breastfeeding, months (±SD)	7.1 (6)	7 (8.5)
Age at schooling, months (±SD)	23.2 (9)	25.9 (8.5)
Siblings, *n* (%)	60 (82.2)	52 (71.2)
*N* of siblings (±SD)	1.4 (0.6)	1.4 (0.6)
Passive smoking, *n* (%)	28 (38.4)	30 (41.1)

**Table 3:** Common infectious diseases observed during the study period.

Disease	Group A	Group B	*p*
Acute gastroenteritis, *n* (%) (number of episodes)	12 (18.2) (19)	24 (40.0) (28)	0.007
Rhinitis, *n* (%) (number of episodes)	22 (33.3) (44)	24 (40.0) (50)	0.438
Otitis media, *n* (%) (number of episodes)	8 (12.1) (11)	13 (21.7) (17)	0.151
Pharyngitis, *n* (%) (number of episodes)	13 (19.7) (22)	25 (41.7) (30)	0.007
Laringitis, *n* (%) (number of episodes)	6 (9.1) (7)	14 (23.3) (14)	0.029
Tracheitis, *n* (%) (number of episodes)	11 (16.7) (16)	19 (31.7) (30)	0.048

## Abbreviations

AGE	acute gastroenteritis
CIDs	common infectious diseases
FPs	family pediatricians
HBD-2	β-defensin
HNP 1-3	α-defensin
LL-37	cathelicidin
sIgA	secretory immunoglobulin
URTI	upper respiratory tract infections
